# Deep learning based automatic segmentation of organs-at-risk for 0.35 T MRgRT of lung tumors

**DOI:** 10.1186/s13014-023-02330-4

**Published:** 2023-08-14

**Authors:** Marvin F. Ribeiro, Sebastian Marschner, Maria Kawula, Moritz Rabe, Stefanie Corradini, Claus Belka, Marco Riboldi, Guillaume Landry, Christopher Kurz

**Affiliations:** 1 Department of Radiation Oncology, LMU University Hospital, LMU Munich, Munich, Germany; 2 German Cancer Consortium (DKTK), Partner Site Munich, Munich, Germany; 3 Bavarian Cancer Research Center (BZKF), Munich, Germany; 4 Department of Medical Physics, Ludwig-Maximilians-Universität München, Garching, Germany

**Keywords:** Deep learning, Auto-segmentation, MR-Linac, MRI-guidance, Thorax

## Abstract

**Background and purpose:**

Magnetic resonance imaging guided radiotherapy (MRgRT) offers treatment plan adaptation to the anatomy of the day. In the current MRgRT workflow, this requires the time consuming and repetitive task of manual delineation of organs-at-risk (OARs), which is also prone to inter- and intra-observer variability. Therefore, deep learning autosegmentation (DLAS) is becoming increasingly attractive. No investigation of its application to OARs in thoracic magnetic resonance images (MRIs) from MRgRT has been done so far. This study aimed to fill this gap.

**Materials and methods:**

122 planning MRIs from patients treated at a 0.35 T MR-Linac were retrospectively collected. Using an 80/19/23 (training/validation/test) split, individual 3D U-Nets for segmentation of the left lung, right lung, heart, aorta, spinal canal and esophagus were trained. These were compared to the clinically used contours based on Dice similarity coefficient (DSC) and Hausdorff distance (HD). They were also graded on their clinical usability by a radiation oncologist.

**Results:**

Median DSC was 0.96, 0.96, 0.94, 0.90, 0.88 and 0.78 for left lung, right lung, heart, aorta, spinal canal and esophagus, respectively. Median 95th percentile values of the HD were 3.9, 5.3, 5.8, 3.0, 2.6 and 3.5 mm, respectively. The physician preferred the network generated contours over the clinical contours, deeming 85 out of 129 to not require any correction, 25 immediately usable for treatment planning, 15 requiring minor and 4 requiring major corrections.

**Conclusions:**

We trained 3D U-Nets on clinical MRI planning data which produced accurate delineations in the thoracic region. DLAS contours were preferred over the clinical contours.

## Introduction

The clinical introduction of magnetic resonance imaging guided radiotherapy (MRgRT) has brought great benefits such as higher dose conformity to the target and more healthy tissue sparing [1]. In contrast to conventional stereotactic body radiotherapy (SBRT) using cone beam computed tomography (CBCT), magnetic resonance (MR) images do not expose the patient to extra dose during image acquisition. The patient’s anatomy in treatment position, as depicted on the daily images, is typically used for online plan adaptation. Real-time cine-MR imaging can also be used for breath-hold gated treatment with high geometric gross tumor volume (GTV) coverage [2]. The MRIdian (ViewRay Inc, Cleveland, OH, USA) [3] is a 0.35 T MR-guided linear accelerator (MR-Linac) that enables such treatments. It requires the target and organs-at-risk (OARs) to be accurately segmented not only on the planning image, but also on the image of the day (fraction image) [4]. Although time is a less critical factor for delineating the planning MR image (MRI), it is still a fairly time consuming procedure [5] and prone to inter- and intra-observer variability [6]. These uncertainties may further affect follow-up analyses, such as dose accumulation studies in the scope of MRgRT [7]. While deformable image registration (DIR) is used in the MRgRT current clinical workflow to propagate contours from the planning image to the daily MRI, such contours often necessitate time-consuming manual correction or re-contouring. Treatment time without irradiation ranges from 30 to 70 min for a single fraction [8–10], of which up to 22 min are due to the delineation [11].

Recent publications have shown that deep learning auto-segmentation (DLAS) can produce accurate delineations, which in turn accelerates the workflow, decreasing patient discomfort as well as increasing patient throughput [12]. Network-generated contours are also more consistent, helping to reduce inter- and intra-observer variability [5]. Several groups have demonstrated that artificial neural networks (ANNs) can produce high quality contours in the context of MRgRT. Liang et al. [13] used a support vector machine based model on pancreatic images from a 0.35 T MR-Linac. Fu et al. [14] have used a convolutional neural network (CNN) with two correction networks to achieve promising results in the abdominal region for the same MR-Linac. Eppenhof et al. [15] used a 3D U-Net to segment the clinical target volume (CTV) for prostate cancer patients by generating a deformation field from the planning image to the fraction image of prostate cancer patients from a 1.5 T MR-Linac. Kawula et al. [16] have demonstrated superior accuracy for prostate and bladder segmentation using a patient-specific 3D U-Net on a 0.35 T MR-Linac. Chen et al. [17] have also used a patient-specific CNN model for prostate cancer patients on a 1.5 T MR-Linac. Fransson et al. [18] used a 2D U-Net model trained from scratch on a single 1.5 T prostate patient planning image. Li et al. [19] used a modified version of nnU-Net 2D [20] for a daily updated patient-specific segmentation of pelvic and abdominal fraction images from a 1.5 T MR-Linac.

Currently, there are few studies on OAR segmentation of thoracic MR images in general (e.g., Dong et al. [21]), none of which are in the context of MRgRT of lung tumors. The goal of this study was to evaluate the performance of DLAS on important OARs for the treatment of lung tumors (lungs, heart, aorta, esophagus and spinal canal) on a 0.35 T MR-Linac. The generated contours were compared to the clinically used ones in a geometrical analysis. They were also graded by a physician with regards to their clinical usability.

## Materials and methods

### Database

This study included data from 112 patients with lung tumors treated at the ViewRay MRIdian MR-Linac installed at the Department of Radiation Oncology of the LMU Munich University Hospital. 122 planning MRIs with their corresponding clinical OAR contours created between January 2020 and September 2022 were retrospectively collected. The patients received fractionated treatment in 3–16 fractions. All patients signed an informed consent form. More details about the patient cohort can be found in Table 1. The MR images were acquired using a 3D balanced steady state free-precession sequence. The images had a $1.5\times {1.5}\,{\hbox {mm}^{2}}$ in-plane resolution in the axial plane with varying axial size (usually $>300\times 300$ pixels) and 3 mm slice thickness, with 144 slices in most cases. Segmentation of the ROIs was performed manually and approved during treatment planning by radiation oncologists. Images were exported from the treatment planning system (TPS) as DICOM files, along with their corresponding contours in DICOM-RT format.

**Table 1:** Summary of details about the entire patient cohort and the training, validation and test set subgroups

Information	Training	Validation	Test	Total
Age				
Median	64	60	64	64
Range	19–86	25–75	29–88	19–88
M:F ratio	48:52	53:47	78:22	60:40
Lesion location				
SL l	17%	37%	9%	19%
SL r	21%	11%	26%	20%
ML r	6%	11%	4%	7%
IL l	17%	5%	30%	18%
IL r	21%	16%	17%	20%
Other	17%	21%	13%	17%
Metastasis	71%	79%	57%	70%

Includes median age and age range (min–max), male to female (M:F) ratio, location of the lesion (SL—superior lobe, ML—middle lobe, IL—inferior lobe, r—right, l—left) and the ratio of metastases to primary tumors. Metastasis denotes lesions of any origin in the lung, as opposed to a primary lung tumor

### Data pre-processing

Contours were converted to binary masks in ITK Meta Image format (mha), using plastimatch [22] with nearest-neighbour (nn) interpolation on the MR reference grid. Binary masks and images were resampled to 1.5 mm slice thickness with nn interpolation using the SITK [23] Python package, resulting in voxels of isotropic dimension. All images were then center-cropped/zero-padded to a uniform size of $256\times 256\times 256$ voxels. Lastly, intensity normalization to values between 0 and 1 while clipping at the image intensity’s 99.5th percentile to account for possible high intensity MR artifacts was applied.

### Network implementation details

The PyTorch based [24] MONAI [25] implementation of a 3D U-Net was used in this study. The network, inspired by Kerfoot et al. [26], had an identical architecture to the one used in Kawula et al. [16]. It has single input and output channels, which are converted to and from 16 channels in the beginning and end. Each following block then doubles the number of channels, from 16 to 256 in four steps with stride 2 down-convolutions in the encoding arm, and does the same in reverse with up-convolutions in the decoding arm. Each residual block consists of 2 series of a convolution, instance normalization and PReLU activation, of which only the first convolution changes the tensor dimensions as described. A Dice similarity coefficient (DSC) based loss function [27] and the ADAM optimizer [28] were used for training. The Dice loss ${\rm{DL}}$ between the network prediction *P* and the ground truth (GT) *Y* was computed as

1 $$\begin{aligned} {\rm{DL}} = 1-\frac{2\sum ^{N}_{i} p_{i} y_{i} +\epsilon }{\sum ^{N}_{i}p_{i}+\sum ^{N}_{i} y_{i} + \epsilon }, \end{aligned}$$

summing over all $N=256\times 256\times 256$ voxels $p_i\in P$ and $y_i \in Y$. The term $\epsilon =10^{-5}$ was used to avoid numerical issues.

Training was performed using an NVidia RTX A6000 (48 GB VRAM) or an NVidia Quadro RTX 8000 (48 GB VRAM) GPU.

**Table 2:** Summary of number of contours used for each set and ROI

OAR	Training	Validation	Test	Sum
Left lung	75	18	20	113
Right lung	78	18	23	119
Heart	74	15	23	112
Aorta	63	18	19	100
Spinal canal	68	18	23	109
Esophagus	80	18	21	119
Total MR scans	80	19	23	122

Numbers differ due to availability of contours in clinical treatment planning

### Training strategy

Planning MRIs were randomly split into 80 training, 19 validation and 23 test MRIs. A separate model was trained for each OAR. Contours were used according to their availability, so if an OAR had not been delineated for treatment, the planning MRI had to be excluded from the model for that particular OAR. The segmented OARs were chosen based on sufficient data availability, which was also seen as an indicator for the most commonly clinically needed segmentations. This led to a final selection of 6 OARs: right and left lungs, heart, aorta, spinal canal and esophagus. The exact split for each OAR is detailed in Table 2.

The network was trained in a two phase process with successive data augmentation. Data augmentation was used to prevent overfitting. During the first phase, it was trained with few computationally inexpensive augmentations and a mini-batch size of 4 for 75 epochs. The second phase was used to improve the DLAS contours by stronger data augmentations. The training continued with the model parameters of the epoch with the highest DSC on the validation set from the first phase. The mini-batch size was decreased to 1 for the second step, and the network was trained for 500 epochs. To further prevent overfitting, the model at the training epoch with the best performance on the validation set was automatically selected retrospectively as the final model for each OAR individually. The aforementioned two step augmentation process starts with translations and rotations, followed by a random zoom and by random Gaussian noise in the first phase. The probability $p_\text {aug}$ for translation, rotation, zoom and Gaussian noise being applied was $p_{\text {1}}$.

The second phase introduced random elastic deformations, MRI motion artifacts and a random MRI bias fields following translation and rotation transformations. For these, the TorchIO [29] Python library was used. All other transformations were implemented using functions from the MONAI library [25]. There was a second intensity clipping of values below 0 and above 1 after the Gaussian noise was applied. For this phase, $p_\text {aug}$ was changed to $p_{\text {2}}$.

### Data post-processing

The network output probabilities were passed through a sigmoid activation function, followed by a threshold of 0.5 to create a binary mask. Connected components with less than $1/8^{\rm{th}}$ of the total volume of the mask were removed before evaluation.

### Data evaluation

#### Geometrical analysis

The DSC and the Hausdorff distance (HD) (average $\text {HD}_\text {avg}$ and 95th percentile $\text {HD}_\text {95}$) were calculated using the clinical contours of the planning MRI as GT. Long, tubular OARs, i. e., the esophagus, spinal canal and aorta were often only delineated in the high dose region near the slices containing the tumor. Thus, these OARs were only evaluated in the axial slices containing the planning target volume (PTV) and the 10 slices above and below.

#### Physician’s grading

A radiation oncologist was presented with the DLAS and the clinical contours of all considered ROIs of the 23 test set MRIs and asked to grade them on a 0–4 scale. These grades represented the following statements: 0—“no clinically relevant correction possible”, 1 — “ready to use”, 2—“minor corrections required”, 3 — “major corrections required”, 4—“unusable”. To reduce possible evaluation bias, the ROI contours were presented as 46 separate MRIs in random order. The radiation oncologist was thus aware that they were comparing DLAS and clinical segmentation, but not which was which. We used the same scale as Kawula et al., but refined it by adding a category 0 to differentiate between contours with clinically acceptable residual errors from those without.

## Results

### Network training

**Table 3:** Functions used during training and hyperparameters that were manually set

Function	Parameter	Tested range	Final value	
			Phase 1	Phase 2
Probability	$p_{\rm{aug}}$	0.25–1	$p_1=0.6$	$p_2=0.85$
Learning rate	*lr*	$10^{-5}$–$2\times 10^{-2}$	$10^{-3}$	
*Spatial*				
Rotation	$\alpha _{\rm{max}}\,[^\circ ]$	5–20	15	
Translation	$\Delta _{\rm{max}}\,[{\hbox {mm}}]$	$15-30$	22.5	
Zooming	$z_{\rm{min}},\, z_{\rm{max}}$	–	$0.9,\,1.1$	
Deformation	$n_{\rm{cp}}$	5–20	–	8
	$d \,[{\hbox {mm}} ]$	15–45	–	24
*MR*				
Motion	$m_{{\alpha }}[^\circ ]$	0–15	–	10
	$m_{\Delta }\,[{\hbox {mm}}]$	15–75	–	45
Bias field	order	1–3	–	1
	$c_{\rm{mag}}$	0–1	–	0.4
Noise	$\sigma$	0.01–0.25	0.05	0.1
	$\mu$	–	0	

Tested range (min–max) and final chosen value for each training phase are given. For more information, refer to documentation of corresponding MONAI or TorchIO functions

The same final set of hyperparameters was used across all OARs. All hyperparameters, aside from $p_{\rm{aug}}$ and the standard deviation of the Gaussian noise were kept unchanged during both training phases. The range of searched hyperparameters can be found in Table 3. The final set includes a learning rate of $10^{-3}$, augmentation probabilities of $p_{\text {1}}=0.6$ and $p_{\text {2}}=0.85$, a standard deviation for the Gaussian noise of 0.05 and 0.1, a zoom range between 0.9 and 1.1, a rotation range of up to $15^\circ$ and translation range of up to 22.5 ${\hbox {mm}}$ in all spatial dimensions. B-Spline elastic deformations used a maximum of 8 control points and a maximum displacement of 24 ${\hbox {mm}}$. MR related augmentations were set to $10^\circ$ and ${45}\,{\hbox {mm}}$ for the motion artifacts and an order of 1 and maximum polynomial coefficient magnitude of 0.4 for the bias field. The bias field from TorchIO was modelled as a unit-less quantity that modifies the voxel intensity by multiplying it with a linear combination of polynomial basis functions with randomly chosen coefficients [30]. The average training duration per epoch was around 90 s during the first phase and 5 min in the second phase.

### Geometric evaluation

**Fig. 1:**
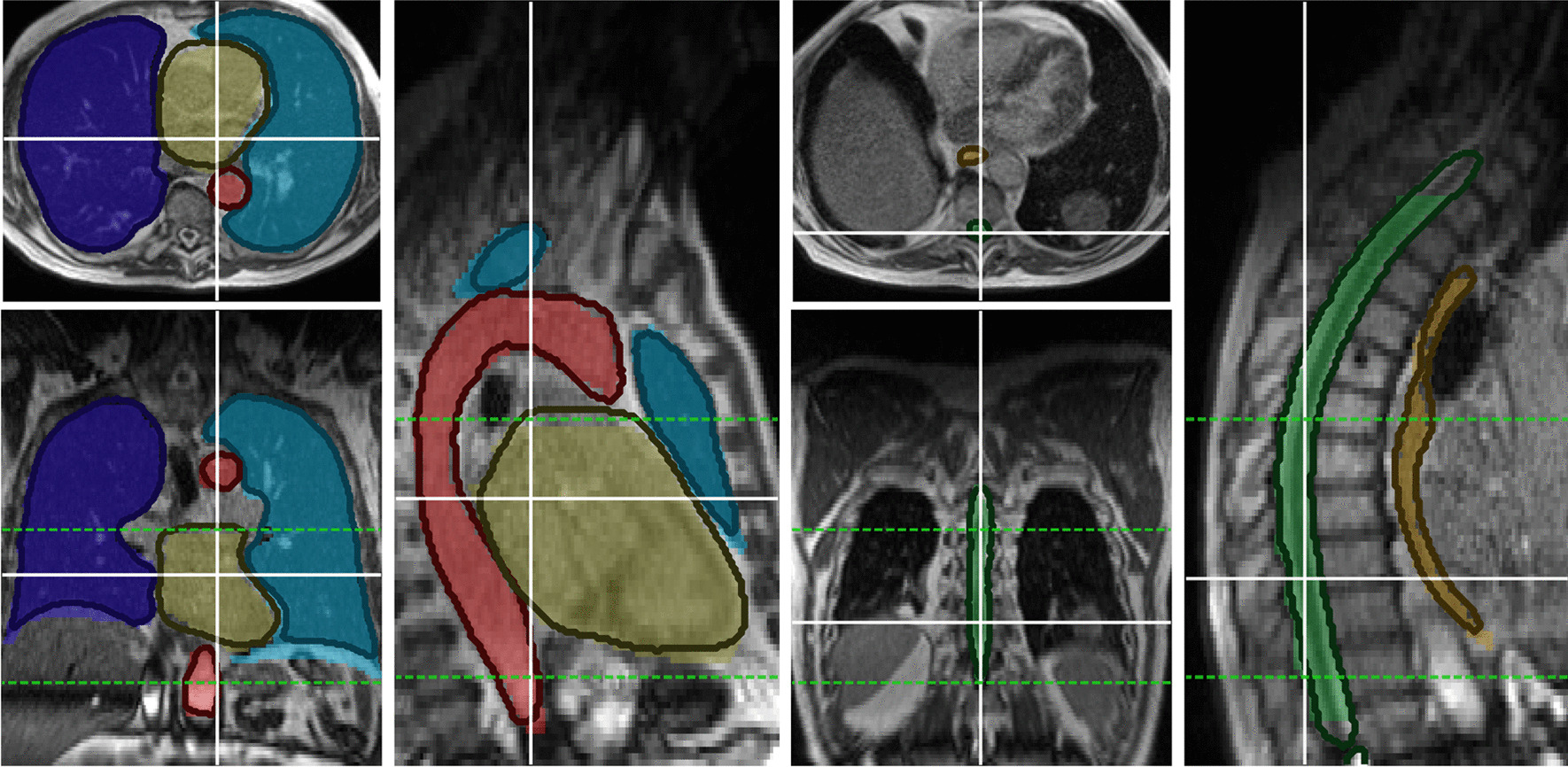
Example DLAS contours (solid outline) and GT contour (colored overlay) in coronal, axial and sagittal view, subdivided into images containing lungs, heart and aorta for the left three panels, and esophagus and spinal canal for the right three panels. The dotted, green lines represent slices containing the PTV ± 10 slices in superior and inferior direction, where the geometrical evaluation of the esophagus, spinal canal and aorta was performed. MR images from a single patient shown

**Fig. 2:**
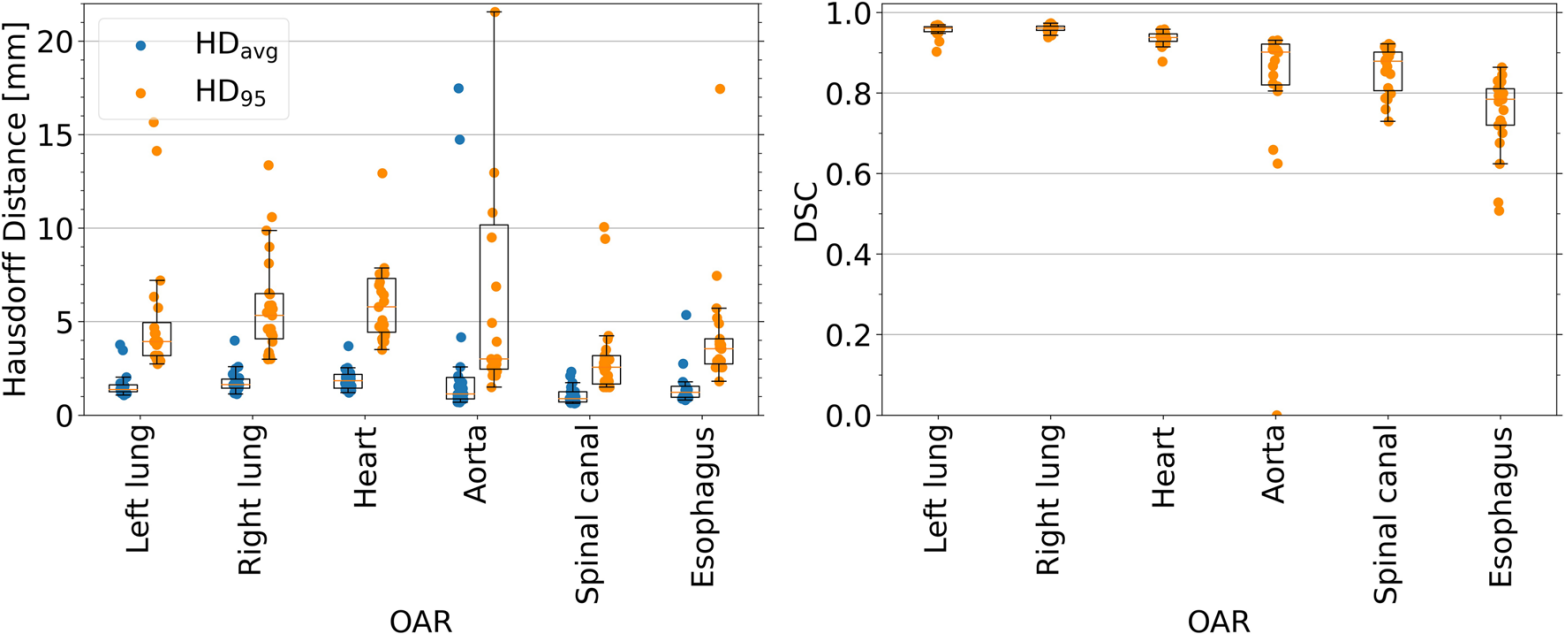
Box plots of Hausdorff distance (left) and Dice similarity coefficient (right) for all test set contours per ROI. Not on display in the left plot are two data points for the ${\rm{HD}}_{95}$ of the aorta at 29.8 ${\hbox {mm}}$ and 37.9 ${\hbox {mm}}$

Figure 1 shows an exemplary MR image with clinical and DLAS contours. Differences in lung and heart contours can be observed on the coronal slices. Differences in length between the spinal canal contours on both ends and the superior end of the heart contour can be observed on the sagittal slices. The chosen axial slices in the high dose region show good agreement between both sets of contours.

DSC for large OARs (lungs and heart) was high with an averaged median value over the three OARs [interquartile range (IQR)] of 0.95 [0.95–0.96]. For the tubular ROIs (spinal canal, esophagus and aorta), DSC was lower with an average median value of 0.86 [0.78–0.88]. The averaged median value of both $\text {HD}_\text {95}$ (5.0 ${\hbox {mm}}$ [3.9–6.2 ${\hbox {mm}}$] vs 3.0 ${\hbox {mm}}$ [2.3–5.8 ${\hbox {mm}}$]) and $\text {HD}_\text {avg}$ (1.6 ${\hbox {mm}}$ [1.4–1.9 ${\hbox {mm}}$] vs. 1.1mm [0.8–1.6 ${\hbox {mm}}$]) was lower for the second group, with the IQR being similar for both groups.

The results per OAR are summarized in Table 4 and visualized in boxplots in Fig. 2.

**Table 4:** Summary of geometrical analysis, showing DSC and HD (95th percentile and average value) for each segmented OAR, median [IQR]

	DSC	${HD}_{95}$	${HD}_\text {avg}$
		(mm)	
Left lung	0.96 [0.95–0.96]	3.9 [3.2–4.9]	1.4 [1.2–1.6]
Right lung	0.96 [0.96–0.97]	5.3 [4.1–6.5]	1.6 [1.4–1.9]
Heart	0.94 [0.93–0.95]	5.8 [4.4–7.3]	1.8 [1.4–2.2]
Aorta	0.90 [0.82–0.92]	3.0 [2.5–10.2]	1.1 [0.9–2.0]
Spinal canal	0.88 [0.81–0.90]	2.6 [1.7–3.2]	0.9 [0.7–1.3]
Esophagus	0.78 [0.72–0.81]	3.5 [2.7–4.1]	1.2 [1.0–1.5]

### Physician’s grading

**Fig. 3:**
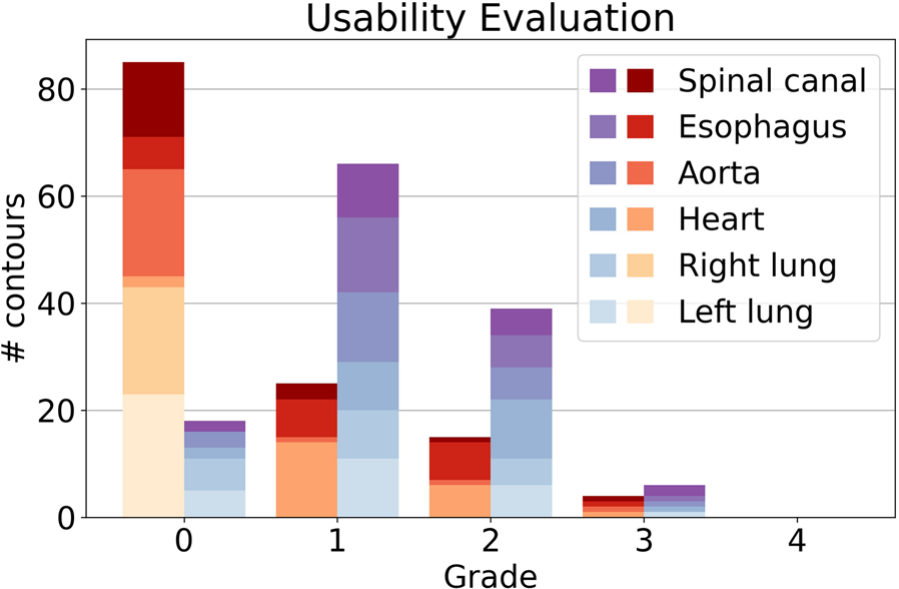
Physician’s evaluation of test set contours (total of 129, see Table 2), subdivided by OARs. DLAS contours on the left in (red hues), clinical contours on the right (blue hues). Grade descriptions: 0—“no clinically relevant correction possible”, 1—“ready to use”, 2—“minor corrections required”, 3—“major corrections required”, 4—“unusable”

The physician’s grading favored the DLAS contours, which were given the rating “0—no clinically relevant correction possible” 85 times, compared to 18 times for clinical contours. This was most apparent for the lungs, the spinal canal and the aorta. The DLAS as well as the clinical contours of heart and esophagus received lower scores than the other OARs on average.

85% of DLAS and 65% of clinical contours were deemed at least “ready to use” (grades 0 or 1). In more detail, 70% and 61% of heart and esophagus DLAS contours and 48% and 67% of the clinical contours respectively received these ratings. Of the remaining OARs (lungs, aorta and spinal canal), 95% of DLAS contours received a grading of “no clinically relevant correction possible” or 1—“ready to use”, versus 69% of clinical contours. A more detailed breakdown can be seen in Fig. 3.

## Discussion

**Fig. 4:**
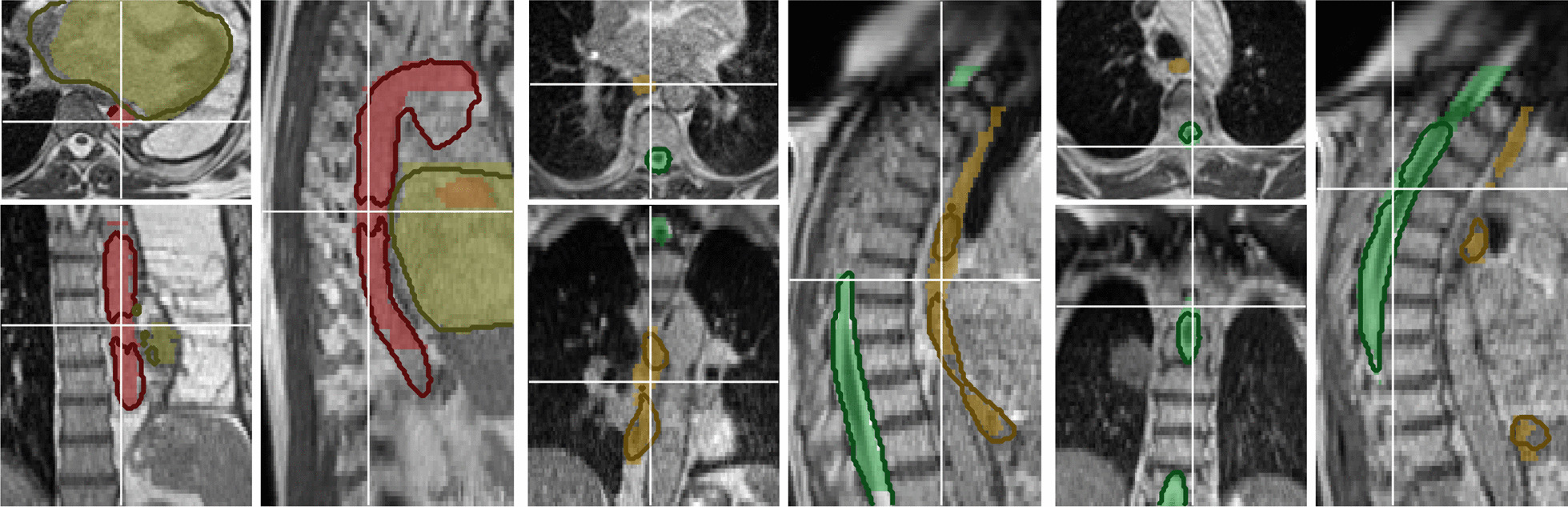
Examples of poor DLAS contours (solid outline) compared to GT contour (colored overlay) in coronal, axial and sagittal view of the aorta (red, left) for P23 and esophagus (orange, middle) and the spinal canal (green, right) for P14

In this study, DLAS for 0.35 T MR-Linac planning images of lung tumor patients was evaluated via geometric analysis by comparing it to the clinically used contours, and via clinical grading by a radiation oncologist. The geometric analysis showed that the DLAS contours were close to the clinically used ones. We achieved reasonably high DSCs for all OARs, which were in line with or better than average (where curated challenge datasets were not used) CT-based DLAS results [31, Table 1] [32]. As mentioned previously, due to the lack of studies on MRI-based DLAS, this was our only basis for comparison. In the majority of cases, the DLAS contours were preferred over the clinical contours by the radiation oncologist.

For both lungs, a DSC of 0.96 was achieved and all DLAS contours received the best grade (0). While high DSC values are more easily achieved by large, high contrast organs, manual lung segmentation is time consuming in the clinical TPS version currently in use due to a lack of automation tools. Our model therefore may lead to substantial time savings. The main differences were attributed to differing contouring styles near the bronchial tree or the tumor, which were included in a few cases, but usually excluded for clinical contours.

The tubular shape of the spinal canal and the aorta result in an increased surface area to volume ratio, and therefore DSCs were lower. Nonetheless, HD was comparable to the lungs. Only 2 out of 23 DLAS contours for the spinal canal and 2 out of 19 for the aorta were deemed to require any kind of correction. These were mostly due to small holes in the structures (grade 3) or some slices only having the OAR partially contoured on one side (grade 2), e.g. due to MRI artifacts. Examples can be seen in Fig. 4.

The average DSC and HD achieved for the heart were comparable to the lungs, which is partly due to a large volume with comparatively small surface area. Grading resulted in 6 out of 23 contours requiring minor corrections, while 1 required major corrections. These were mainly due to the heart wall not being included in the DLAS contour.

As with previous studies on CT images by other groups [31, Table 1] [32], average DSC scores of the esophagus contours were worse than for all other OARs. The poor contrast to the surrounding tissue and possibly decreased sharpness due to abdominal motion, make it difficult to segment for both physicians and the network. The much larger variety in possible shapes in the axial slices and differing lengths of the contour in the training data are also possible reasons for the worse performance for this OAR. Other groups such as Fu et al. [14] reported similar problems with the duodenum, which behaves similarly to the esophagus in this context.

Segmentation on patients with an uncommon anatomy, such as a collapsed or removed lung, were also included (1 in training, 1 in validation, 2 in the test set, P18 and P23). DLAS contours of test case P18 received a near perfect score (grade 0 for right lung, spinal canal, esophagus and aorta, grade 1 for heart), whereas test case P23’s DLAS contours received the worst grading overall (grade 0 for right lung and spinal canal, grade 2 for heart and esophagus, grade 3 for aorta).

In Fig. 2, five cases stand out (aorta segmentations with DSC of 0.00, 0.63 and 0.66, P3, P21 and P23 respectively, and esophagus segmentations with DSC of 0.51 and 0.53, P13 and P14 respectively). The largest deviation in DSC was the aorta segmentation for P3 with a DSC of 0 and a ${\rm{HD}}_{95}$ of 30 ${\hbox {mm}}$. In this case, the ground truth contour was only done in the inferior part of the MRI, despite the target region being located in the superior part. The physician’s grading of this patient was 0 for the DLAS and 3 for the ground truth. Similarly, P21 had a DSC of 0.63 and a ${\rm{HD}}_{95}$ of 38 ${\hbox {mm}}$ for the aorta, but was graded with 0 for DLAS, and the ground truth was graded with 1. The poor DSC and HD were due to the network fully segmenting the aorta, while the clinical contour did not include the ascending aorta and the arch of the aorta, since they were farther away from the tumor. Excluding these cases would lead to median [IQR] values of 0.91 [0.84–0.92], 2.9 [2.4–6.9]  ${\hbox {mm}}$, 1.0 [0.9–1.7]  ${\hbox {mm}}$ for DSC, ${\rm{HD}}_{95}$ and ${\rm{HD}}_{\rm{avg}}$ respectively for the aorta. The aorta DLAS contour of P23 had a DSC of 0.66 and a a ${\rm{HD}}_{95}$ of 22 ${\hbox {mm}}$ and received a grade of 3. In this case, the DLAS failed by including a sizable part of the heart in the segmentation, exhibiting a minor hole and not continuing the contour far enough into the heart. The esophagus DLAS contours had two cases with large deviations. P13’s DLAS contour was however graded with 0, compared to the ground truth’s 1, despite the DSC of 0.51 and ${\rm{HD}}_{95}$ of 17 ${\hbox {mm}}$. For P14, DSC and ${\rm{HD}}_{95}$ were 0.53 and 7.4 ${\hbox {mm}}$ respectively. The DLAS contours received a grading of 3 and the ground truth a grading of 1. Here, the DLAS contour exhibited a hole in the middle of the contour.

In these cases, it can be concluded that the contour quality is not always well reflected by the DSC and HD. A geometric analysis using clinical data as the ground truth has its limitations, as evidenced by Vaassen et al. [33]. For example, a segmentation with a good geometric evaluation can still lead to low gamma pass rates when used for treatment planning, as indicated by Kawula et al. [34]. The grading system was found to be more helpful at gauging the quality of DLAS contours for the purpose of this study. We also acknowledge that the physician might look at the contours differently in a review compared to a treatment setting. Clinical contours are generated during treatment workflows, and the physicians tend to focus on the high dose region around the lesion, which is most relevant for treatment planning. Clinical contour quality farther away from the treatment region may thus be lower.

This appears to not have noticeably hindered training, as most deviations appear to even out in DLAS models, given enough training data. Lustberg et al. [35] have also found that using models trained on non-curated local data could still save 50% time compared to manual contouring. Nonetheless, the variations in length of the esophagus, spinal canal and aorta contours remain a challenge. We used masks to only consider the tumor region when selecting the best model during validation, but did not take any measures to alter the training process in this regard. This was intentional, as any clipping of the masks would result in DLAS contours being shorter in general. We judged that, in a scenario where these contours are presented to a physician as a starting point, it would be more time efficient for them to remove or ignore distal, inaccurate parts, as opposed to having to expand a contour that is too short. However, we suspect that the inconsistent length of these contours in the training data might be a contributing factor for the occasional holes in these OARs. A more uniform training set, acquired by re-contouring the training data, could lead to some further segmentation improvements for the underperforming OARs (esophagus and heart). Similarly, patient specific model fine-tuning with a single training patient would likely create more consistent contours (as demonstrated by Kawula et al. [16]). Albeit that would only apply to the fraction images, as opposed to new planning images.

The goal of automatic segmentation is to help physicians with delineating structures. This means reducing the time spent manually delineating structures and decreasing observer variability. Evaluation methods need to be chosen with these aspects in mind. The DLAS contours should therefore not perfectly fit the existing ground truth, but rather require as few corrections as possible. In a next step, we will quantify the time saved in the MRgRT workflow by prospectively providing physicians with these contours for OAR delineation in treatment planning [36, 37].

## Conclusion

In conclusion, we trained U-Nets for contouring the lungs, heart, aorta, spinal canal and esophagus on thoracic images from an 0.35 T MR-Linac. They were able to produce contours that were most of the time preferred to the clinical contours by a radiation oncologist.

## Data Availability

The data used in this study cannot be made available due to data protection regulations.

## Abbreviations

MR: Magnetic resonance
MRI: Magnetic resonance image
MRgRT: Magnetic resonance (imaging) guided radiotherapy
DLAS: Deep learning autosegmentation
DSC: Dice similarity coefficient
HD: Hausdorff distance
SBRT: Stereotactic body radiotherapy
CBCT: Cone beam computed tomography
GTV: Gross tumor volume
MR-Licac: MR-guided linear accelerator
OAR: Organ-at-risk
DIR: Deformable image registration
ANN: Artificial neural network
CNN: Convolutional neural network
CTV: Clinical target volume
TPS: Treatment planning system
mha: Meta image format
nn: Nearest neighbour
${\rm{HD}}_{\rm{avg}}$: Average Hausdorff distance
${\rm{HD}}_{95}$: 95th percentile Hausdorff distance
GT: Ground truth
PTV: Planning target volume
IQR: Interquartile range
